# The Reck System of Heating

**Published:** 1912-03-09

**Authors:** 


### The Reck System of Heating.

The advantages claimed for this system, which is named after the Danish engineer who invented it, are that it can be combined with almost any system of ventilation, that the cost of installation and of upkeep is less than that of an ordinary hot-water system, and that by means of regulators, the heat in the various rooms or portions of the same building can be altered at will. In addition, both heating and cooling can be effected very quickly.

In the Reck system a constant and rapid flow of water is ensured by means of a circulator, between which and the radiators flow and return pipes are fixed in much the same way as in an ordinary gravity system. The water is partially heated by low-pressure steam in a "re-heater," to which the flow and return pipes are also connected. Above the highest radiator an expansion tank is fixed, and to this the water in the flow pipe is taken before beginning its work of heat distribution through the building. A few feet below the expansion tank is fitted (on the flow pipe) the patent circulator. Through this low-pressure steam is forced, and this causes the water in the pipe, between the circulator and the expansion tank, to boil. A condenser is fitted on the ascending flow pipe just below the circulator, and the steam discharged above the water line in the expansion tank is condensed by the partially heated water coming from the re-heater. The over-balancing effect obtained by the difference in weight between the water mixed with steam in the ascending flow pipe, and the water without steam in the same length of descending flow pipe, is sufficient to cause a circulation about four times as fast as that obtained in an ordinary gravity system.

This rapid circulation ensures the quick heating and cooling to which reference has already been made, and it also renders it possible to use supply piping of much smaller bore than has hitherto been required. However unfavourably the radiators are placed, whether by distance from the heater, lack of height over it, or even should they be placed below the return pipe, their fullest efficiency is secured. In addition, radiators 10 or 15 per cent. smaller than is usual may be relied on to give a perfectly satisfactory result. By this means a reduction of about 40 per cent. in the amount of water in the system, from that required in an ordinary gravity system, is obtained. There is no waste in fuel consumption, and the steam which accelerates the circulation at the same time heats the water in the circulator and the condenser.

A pressure of from 3 to 6 lb. is required, and may be obtained from high-pressure boilers and reduced, or if the hospital has separate pavilions a low-pressure boiler may be installed in each. Any temperature between 100° F. and 185° F. may be obtained, but the piping is usually designed to give a mean pressure in the radiators of 185° F. when maximum work is required from the apparatus. Sectional regulators may also be fixed, and each radiator may be controlled by valves on the inlet and outlet connections.

The cost of installation and upkeep will also be found to compare favourably with that of other systems. We are indebted for the above particulars to Dr. Mackintosh's book on the "Construction, Equipment, and Management of a General Hospital."

